# Navigating data standards in public health: A brief report from a data-standards meeting

**DOI:** 10.7189/jogh.14.03024

**Published:** 2024-04-05

**Authors:** Heather Hufstedler, Yannik Roell, Andressa Peña, Ankur Krishnan, Ian Green, Adriano Barbosa-Silva, Andreas Kremer, Clair Blacketer, Isabel Fortier, Kit Howard, Bess LeRoy, Eza Hafeza, David Baorto, M Swertz, Lauren Maxwell, Thomas Jaenisch

**Affiliations:** 1 Heidelberg Institute of Global Health (HIGH), Heidelberg University Hospital, Heidelberg, Germany; 2 Center for Global Health, Colorado School of Public Health, Aurora, Colorado, USA; 3 Starlz Transplant Institute, School of Medicine, University of Pittsburgh, Pittsburgh, Pennsylvania, USA; 4 SNOMED, SNOMED International, One Kingdom Street, Paddington Central, London, England, UK; 5 ITTM, i2b2 tranSMART, ITTM S.A. (Information Technology for Translational Medicine), Luxembourg; 6 Janssen Research and Development, Janssen Research & Development, Raritan, New Jersey, USA; 7 Maelstrom, Research Institute of the McGill University Health Centre, Montreal, Quebec, Canada; 8 CDISC, Austin, Texas, USA; 9 LOINC, Regenstrief Institute, Indianapolis, Indiana, USA; 10 EU-CAN-CONNECT, Faculty of Medical Sciences, University of Groningen, Antonius Deusinglaan, the Netherlands

Data standardization offers significant benefits for industry and regulators alike, suggesting that it should be easy. In practice, however, the process has been hard and slow moving. Moving from an abstract incentive-based analysis to one focused on institutional detail reveals myriad frictions favoring the status quo despite foregone gains *– Richard Berner and Kathryn Judge [**1**].*

Data standardisation is not merely a technicality but a fundamental cornerstone in the field of public health. It plays a pivotal role in enabling effective data sharing, pooling, analysis, and interpretation, thereby facilitating informed decision-making during infectious disease outbreaks [2]. Despite its undeniable significance, the journey towards achieving data standardisation has been challenging and slow. Even the term ‘data standardisation’ is ambiguous, as it can have a variety of meanings – e.g. there are standards for new data collection, data storage and analysis, and for mapping existing data to a standard. While these example definitions are closely linked – in that each process focuses on the organisation, or homogenisation, of data – this paper will focus on explaining data standardisation tools for the three primary research steps: collection, storage and analysis.

What is meant by clinical data standards for collection, storage and analysis is often ambiguous, as many standards cross lines in their functions. For example, standards such as the Systematized Nomenclature of Medicine Clinical Terms (SNOMED), Health Level Seven (HL7) Fast Health Interoperability Resources (FHIR), Logical Observation Identifiers Names and Codes (LOINC), Digital Imaging and Communications in Medicine (DICOM), International Statistical Classification of Diseases (ICD), Clinical Data Interchange Standards Consortium (CDISC), Observational Medical Outcomes Partnership (OMOP), Informatics for Integrating Biology and the Bedside (i2b2) transSMART, RxNORM may be collectively recognised by some in the field as falling under the broad umbrella of ‘clinical data standards’. However, each has its own specific focus: OMOP, for example, is dedicated to observational database research, whereas CDISC has generally focused on providing frameworks for collection, reporting, and submission of clinical trial data to governmental regulatory bodies, like the Federal Drug Administration (FDA) in the USA.

In light of these challenges and opportunities, some of the most relevant data standards organisations to international health care organisations were invited to participate in a virtual meeting organised by Reconciliation of Cohort Data in Infectious Diseases (ReCoDID) in March 2022 [2], which was focused on data standardisation for observational infectious disease studies. The standards were chosen for their widespread adoption by international health organisations and pivotal roles in enhancing data quality and interoperability. This paper focuses on the five standards organisations who attended the virtual meeting – OMOP, LOINC, SNOMED, CDISC, and i2b2. Later on in the discussion, we will briefly address the concepts of data harmonisation and the role of data catalogues in supporting these efforts.

By focusing on these selected standards and related themes, we aim to enhance the recognition and understanding of data standardisation among academic researchers, as well as highlight the diverse yet interconnected landscape of data standardisation tools that underpin modern health care research and practice. The ultimate goal is to encourage broader adoption and more informed application of these standards, thereby enriching the global and public health field.

## WHY DATA STANDARDISATION MATTERS IN PUBLIC HEALTH

### The power of data pooling

Pooling data from multiple sources is a cornerstone of robust population health research. It not only enhances the statistical power of studies but also enables efficient resource utilisation by avoiding the redundancy of conducting large-scale studies [3,4]. Data standardisation simplifies the process of harmonising data collected across different studies, making it easier to integrate and analyse, ultimately driving evidence-based public health decision-making.

### Challenges in data harmonisation

Retrospective data harmonisation, the process of reconciling data collected in non-standardised ways, can be resource-intensive and time-consuming, often taking several years before researchers can publish their findings [5,6]. Clinical data elements collected during research studies may be tailored to the specific needs of the investigators, leading to variations in wording, units, or formats. Data standardisation addresses these challenges by facilitating semantic interoperability, a critical element in adhering to the FAIR (findability, accessibility, interoperability, and reusability) principles. Clinical data elements collected during research studies may be tailored to the specific needs of the investigators, leading to variations in wording, units, or formats. Data standardisation addresses these challenges by facilitating semantic interoperability, a critical element of in adhering to the FAIR [7].

### Main data standards in public health

The landscape of data standards in public health is diverse, with numerous standards focusing on different aspects of data, including collection, storage, and analysis. This article highlights five major data standards and organisations that play pivotal roles in the harmonisation of health data. The following information summarises presentations given by these organisations during a virtual conference organised by ReCoDID in March 2022 [2].

#### Clair Blacketer on behalf of OMOP

The Observational Medical Outcomes Partnership (OMOP) was a public-private partnership initiated in 2009 to inform the appropriate use of observational health care databases for studying the effects of medical products. While OMOP itself closed in 2014, the common data model (CDM) and Standard Vocabulary live on as the foundation for large-scale, standard analytics developed by the Observational Health Data Sciences and Informatics (OHDSI) open collaborative, based in the Department of Biomedical Informatics at Columbia University. Over the years the CDM has evolved to better represent observational health data in ways that can easily be leveraged to generate evidence. Some recent changes to the CDM focus on storing variables for natural language processing research and updating demographic variable information related to race and ethnicity.

OMOP is being used in 74 different countries, including the European Health Data and Evidence Network (EHDEN) which includes 140 different data partners across Europe. Recently, the Data Analysis and Real-World Interrogation Network (DARWIN) EU project was initiated by the European Medicines Agency to help them make informed decisions on drug outcomes using observational data, all of which have been standardised to the OMOP CDM. In the USA, the National COVID Cohort Collaborative, funded by the National Institutes of Health, combines COVID data from a system of hospitals to support COVID research, all using the OMOP CDM.

The OHDSI collaborative is open-source, not pharma funded, and international. During extraction, transformation, and load processing of converting data to the OMOP CDM, the data structure, conventions, and content are all standardised while preserving the source data. The structure of the CDM and how to implement the current version are described in detail on the working groups GitHub page [8]. Unique in its approach, the standardised vocabulary is used to map source codes like ICD10, ICD10CM, Read, CPT4, etc. to one common standard. This allows research to be conducted in a federated way because not only is the structure standardised in the tables and fields but the content is standardised in the use of the same ontologies. These vocabularies drive domain and data movement to bring together databases from around the world. A large set of open-source software and packages have also been developed to work with the OMOP CDM since the structure and content are standardised.

#### Eza and David on behalf of LOINC

Logical Observation Identifiers Names and Codes (LOINC) was started in 1994 by Clem McDonald who recognised the need for a universal language for clinical observations due to the trend of sending electronic clinical data from laboratories to others using the data for care and management purposes. The LOINC Committee, organised by Indianapolis-based non-profit medical research Regenstrief Institute, associated with Indiana University, developed a common language for laboratory and clinical observations to help with this issue. Since the observations and measurements that are recorded as part of laboratory test results still tend to contain local and institution-specific codes that are difficult for a receiving external care institution to decipher seamlessly, LOINC provides universal codes for identifying tests and observations. Originally narrowly focused on laboratory needs, the acronym stood for Laboratory Observation Identifiers Names and Codes. As the scope of the standard expanded into domains other than laboratories, the L in the acronym changed to Logical.

LOINC is currently used in over 180 countries, and works closely with federal policy makers, like the Food and Drug Administration (FDA) on creating manuals to map the LOINC codes for In-Vitro Devices (IDD) devices. Collaborations also include the Office of National Coordinator for Health Information and Technology, the National Library of Medicine, and many more. LOINC also currently collaborates with HL-7 on projects for the American Medical Informatics Association and the WHO’s International Classification, and the GIC Joint Initiative Council.

LOINC is supported by both private and public federal funding from the Regenstrief Institute, OMC, the NLM, the FDA and others to provide a service worldwide at no cost. The standard has 98 000 laboratory clinical order and results concept variables, and is available in 19 linguistic variants in 12 languages. There is a clinical committee with nursing, document ontology and radiology subcommittees. Education and training are offered to people who wish to use the standard. Videos and articles documenting the latest research are posted on the LOINC website to aid in learning the basics and showing what updates are being conducted. In addition to the learning material from the website, there are webinars to inform users of any specific updated content being released or technical improvements and changes in the tooling and processes.

#### Ian Green on behalf of SNOMED

The Systematized Nomenclature of Medicine Clinical Terms (SNOMED CT) was originally developed by the College of American Pathologists (CAP) in collaboration with the UK NHS. The terminology was developed by combining SNOMED Reference Terminology – SNOMED RT (CAP) and Clinical Terms V3 – CTV3 (NHS). SNOMED developments through CAP had focused predominantly on pathology, whilst CTV3 was developed predominantly to support primary care. In 2007, NHS and CAP signed over their rights to SNOMED CT to the International Healthcare Standards Development Organization (IHTSDO). The IHTSDO changed its trading name to SNOMED International, and SNOMED CT is no longer referred to as Systematized Nomenclature of Medicine Clinical Term.

SNOMED CT is being used in over 80 countries globally. SNOMED International is a membership organisation, which brings together 48 countries who are responsible for guiding the development of SNOMED CT. The standard was developed using formal description logic, clinical use case requirements and linking to existing clinical knowledge resources where available. SNOMED CT is customisable using extensions, subsets, maps (e.g. ICD-10, ICPC-2, LOINC), and language preferences. The different language preferences make SNOMED the largest multilingual clinical terminology currently available. Further, SNOMED International has formed collaborations with industry, standards organisations and international clinical bodies.

SNOMED International is a not-for-profit organisation that maintains, distributes, and licenses SNOMED CT. As a membership organisation, SNOMED CT is owned by the SNOMED International members. The countries have equal partnerships, with membership payments based on the country’s GDP. Once a member country has purchased a license, anyone in the country is permitted to use it – individual vendors, hospitals, researchers, academic institutions. If someone is interested in using it for humanitarian, research, or development purposes but does not have access to it through a country license, a free license may be applied for and granted. With the entire workforce being virtual and not in brick-and-mortar, overhead cost is reduced and the financial contributions are used solely to maintain and update the product. To make changes to the standard there are online request submission portals, with a central portal that takes requests solely from national centres, then each country has its portal to manage national requests. Any clinician who requires a change to SNOMED CT, may request a change through these mechanisms. SNOMED International develops its own software, which is made available as open source, using an APACHE 2 license. This means that there are tools for mapping within their standard, in addition to a large GITHUB community supporting and developing these tools [9].

#### Kit Howard on behalf of CDISC

Approximately 23 years ago, CDISC developed a standard structure (called SDTM) for representing data that was collected in clinical research trials, to prepare them to be submitted to the US FDA, and for several years this was CDISC’s only standard. Since then, additional standards have been added to span the drug development process, including data collection (CDASH), analysis (ADaM), controlled terminology, data transfer (ODM), and others. Initially, the primary focus of CDISC standards was initially prospective clinical and select non-clinical research intended for regulatory submission and review, but over the last five years CDISC has been working on a number of initiatives to make the standards more flexible and better-suited for retrospective as well as observational and registry data.

CDISC standards have a broad application across academic, public, and private sectors. While it is a requirement to submit pre-clinical and clinical data in CDISC format to regulatory bodies such as the US FDA and Japan’s Pharmaceuticals and Medical Devices Agency (PDMA), the actual usage of CDISC standards spans a much wider array of entities. This includes pharmaceutical companies, biotechnology firms, contract research organisations (CROs), academic research institutions, and health care providers, whose adoption of CDISC standards makes it easier to pool (or merge) data from several studies for future analysis. In addition, the European, Chinese, and South Korean authorities encourage the adoption of CDISC standards.

Usage of CDISC standards is free to anyone. Membership rates (which supports the organisation in its work and allows access to additional tools, products and services) vary [10] depending on the size of the organisation. Academic institutions, government agencies, and NGOs receive special rates. CDISC has developed six separate standards: PRM (protocol), CDASH (data capture), SDTM (aggregation), ADAM (analysis), ODM (transport and archive), and Define-XML (metadata), as well >45 therapeutic area standards. These CDISC standards are based on controlled terminology, and each of them uses domains, which are collections of data elements with similar characteristics grouped around a topic. This helps to provide structure to capture and analyse the data, as well as the capture of data, so there is an integrated flow throughout the study. Recent efforts have started an open source tool platform to leverage the existing communities’ knowledge and expertise. Users who either would like assistance in creating, or have already created, open source tools that might be of use to the larger community can now make them available through the CDISC Open-Source Alliance (COSA) [11].

#### Andreas Kremer and Adriano Barbosa on behalf of ITTM (and i2b2)

The Information Technology for Translational Medicine (ITTM) incorporated in 2015 but is building on work which started back in 2005, when i2b2 was founded through the NIH-funded Harvard Medical Center Project. In 2016, i2b2 became a non-profit foundation. Another organisation, tranSMART, was developed in 2009 by scientists at Johnson & Johnson and Recombinant Data Corporation. The tranSMART foundation was founded in 2013. The two foundations merged into what is now i2b2 tranSMART in 2017, which works in the fields of data management, data curation, using standards to harmonise and accurate pharma data, preclinical, clinical study data.

Over 500 hospitals, research centres, industry and academic institutions are using the software at the time of publishing this paper.

It is an ontology-driven architecture with community-developed plugins and interfaces that enable a variety of configurations to support the needs of data scientists, academic and clinical investigators, and industry. i2b2 and tranSMART are modular open-source software for query, exploration and analysis of clinical, translational and genomics data. ITTM also works with other organisations (e.g. OHDSI, EHDEN) to provide data management and curation services.

### Things to consider when selecting a standard

The concept of data standards has many definitions, depending on the individual or organisation creating the standard. The structures of each can vary drastically. For example, the terms ‘domains’ or ‘axes’ may have different meanings in different standards. One initial hurdle to retrospective standardisation is that data are often already created in a quasi-standard. A second hurdle is that a researcher may have dozens of standards to choose from (e.g. Unified Medical Language System [12] list of vocabularies from other organisations), and does not know where to begin to outline criteria to select a standard. A third issue may arise once mapping to the selected standard begins, when, perhaps, only part of the data collected is compatible with parts of the selected standard.

There are many considerations when choosing a standard. For example, will the data consist of both patient-centric data (e.g. demographics, past medical history) and higher dimensional data, such as -omics related data (e.g. genome, transcriptome, metabolome) or only consist of one or the other? What is the end goal for the data, for example, pooling, analysis, publication, repository? Does the data need to be submitted to a specific regulatory body to fulfil funding requirements? If yes, the regulatory body will have guidelines of which standard must be used. For example, any data being submitted to the Federal Drug Administration in the USA or the Pharmaceuticals and Medical Devices Agency in Japan needs to be compliant with CDISC.

Once a standard has been implemented within the data, it does not necessarily mean the data cannot be transformed into a different standard. There is no all-in-one data standard, and, as this paper has highlighted, there are benefits and drawbacks of each data standard. Pooling individual patient-level data are a vital activity in population health research, and when data are pooled from multiple studies recorded in different standards composed of diverse semantic foundations, interoperability between standards is crucial. Therefore, it is challenging to know their true interoperability but the landscape of how these standards are connected is vital to understand (**Table 1**). OMOP is interoperable with CDISC, SNOMED, and LOINC. Recent efforts have focused on harmonising the models between OMOP-OHDSI and SNOMED. For data capture, LOINC is working with OMOP-OHDSI, SNOMED-CT, among others. SNOMED and CDISC are open source data standards.

**Table 1:** Current state of interoperability among the standards

	OMOP	LOINC	SNOMED	CDISC	i2b2 tranSMART
**OMOP**	X	X	X	X	X
**LOINC**	YES	X	X	X	X
**SNOMED**	YES	YES	X	X	X
**CDISC**	YES	YES	NO	X	X
**i2b2 tranSMART**	YES	YES	NO	YES	X

Some efforts have focused on creating a ‘click-n-pick’ tool to aid users in making an informed decision about which standard and database to utilise. FAIRsharing [13] is one such tool designed to aid users in mapping relationships among standards, policies and databases. This online tool offers information on 1720 standards, 2095 databases, and 174 policies across a wide range of subjects, including natural sciences (e.g. biomedical, chemistry, astronomy, agriculture, earth sciences and life sciences), engineering, and humanities and social sciences (please note that FAIRsharing website is constantly being updated, and that the numbers we claim above were confirmed on 5 January 2024). While a tool of this magnitude is beneficial for new standard users since most of the relevant information is located in one place, issues arise with the accessibility to new users and how current the list within the tool is. The information provided on how to use the tool is not conducive for beginners and due to the overwhelming amount of choices makes it difficult to narrow down for first-time users. Maintenance of such a large catalogue of information may prove difficult since the list needs to be updated regularly due to the changing landscape of the standards.

Related to these standardisation efforts is the work of EUCAN-CONNECT and Maelstrom, both leaders in data harmonisation. Maelstrom has published guidelines for retrospective data harmonisation [14], is available to hire for harmonisation efforts, and also has a catalogue [15] of – at the time this article is published – 139 individual studies and nearly 1.5 million variables. Maelstrom has recently collaborated with the European Bio-Bank in the BioSHARE project [16], retrospectively harmonising (or, mapping) eight population-based studies in six European countries.

Similar to Maelstrom, EUCAN-CONNECT focuses largely, but not solely, on enabling integrated analysis of human data from consortia or multi-centre studies. To do this, the group has two main objectives; first, to improve the way that this heterogenous data are catalogued, and, second, to implement federated analysis using this data. Similar to Maelstrom’s catalogues, EUCAN-CONNECT manages about 20 catalogues of participating studies and their variables so that interested parties may evaluate whether they may prospectively harmonise with those variables. EUCAN-CONNECT has bridged EU data harmonisation consortia with Maelstrom, which is also described in a catalogue of similar standards [17].

### Helping studies adopt standards

All organisations included in this paper are taking steps to improve, whether it be expanding their vocabulary or strengthening (or embarking on) collaborations with other data standards to improve translatability. CDISC, for example, which was originally focused on data from randomised trial designs intended for regulatory submission, is working on a project to create a start-up pack aimed at studies – interventional or observational – not intended for regulatory submission, as well as creating a more user-friendly implementation guide.

Through a project called Fair Plus [18], ITTM and other standard organisations within the EU network are facilitating harmonisation of data. LOINC is attempting to integrate more infectious disease terminologies. SNOMED is working on two projects to make its standard more user-friendly for infectious disease studies: one is looking at the representation of organisms currently in SNOMED and linking that to industrial databases like MALDI TOF (Matrix-assisted Laser Desorption Ionization – Time of Flight), or academic terminologies, which are more research-oriented, and trying to come up with a reasonable representation; the other project is through UNICAM, doing a huge overhaul of the drug content within SNOMED. Additionally, SNOMED International is working with several international groups to assess SNOMED CT usage in supporting national natural language processing, and is also working with the International Collaboration on Cancer Reporting (ICCR) to codify the elements within synoptic reports.

## CONCLUSIONS

In conclusion, the journey towards data standardisation in public health, as discussed in this report, underscores both the complexity and the critical necessity of adopting universal data standards like OMOP, LOINC, SNOMED, CDISC, and i2b2 tranSMART. We hope that this brief report increases awareness, at least in small part, among researchers of what standardisation is, the benefits of adopting a standard, and assists in finding a suitable standard. Despite the challenges presented by diverse data formats and the intricacies of harmonising data across multiple domains, the collaborative efforts and insights shared during the ReCoDID virtual meeting illuminate a path forward. By fostering great understanding and use of these standards, we can significantly enhance data quality, interoperability, and the efficiency of public health research. The discussions underscore the importance of collaboration among international health care organisations, researchers, and data scientists to overcome barriers and leverage these standards for the betterment of global health outcomes. As we continue to navigate the complexities of data standardisation, the collective commitment to improving public health data will undoubtedly pave the way for more informed, effective and timely responses to global health challenges.
